# Developmental Programming of Renal Function and Re-Programming Approaches

**DOI:** 10.3389/fped.2018.00036

**Published:** 2018-02-27

**Authors:** Eva Nüsken, Jörg Dötsch, Lutz T. Weber, Kai-Dietrich Nüsken

**Affiliations:** ^1^Pediatric Nephrology, Department of Pediatrics, Medical Faculty, University of Cologne, Cologne, Germany

**Keywords:** kidney development, nephron number, renin–angiotensin–aldosterone system, renal sodium transport, blood pressure, early nutrition, re-programming intervention

## Abstract

Chronic kidney disease affects more than 10% of the population. Programming studies have examined the interrelationship between environmental factors in early life and differences in morbidity and mortality between individuals. A number of important principles has been identified, namely permanent structural modifications of organs and cells, long-lasting adjustments of endocrine regulatory circuits, as well as altered gene transcription. Risk factors include intrauterine deficiencies by disturbed placental function or maternal malnutrition, prematurity, intrauterine and postnatal stress, intrauterine and postnatal overnutrition, as well as dietary dysbalances in postnatal life. This mini-review discusses critical developmental periods and long-term sequelae of renal programming in humans and presents studies examining the underlying mechanisms as well as interventional approaches to “re-program” renal susceptibility toward disease. Clinical manifestations of programmed kidney disease include arterial hypertension, proteinuria, aggravation of inflammatory glomerular disease, and loss of kidney function. Nephron number, regulation of the renin–angiotensin–aldosterone system, renal sodium transport, vasomotor and endothelial function, myogenic response, and tubuloglomerular feedback have been identified as being vulnerable to environmental factors. Oxidative stress levels, metabolic pathways, including insulin, leptin, steroids, and arachidonic acid, DNA methylation, and histone configuration may be significantly altered by adverse environmental conditions. Studies on re-programming interventions focused on dietary or anti-oxidative approaches so far. Further studies that broaden our understanding of renal programming mechanisms are needed to ultimately develop preventive strategies. Targeted re-programming interventions in animal models focusing on known mechanisms will contribute to new concepts which finally will have to be translated to human application. Early nutritional concepts with specific modifications in macro- or micronutrients are among the most promising approaches to improve future renal health.

## Introduction

Prevention of chronic kidney disease is a major public health challenge (1). Although diabetes mellitus is the most common cause of chronic kidney disease worldwide (2), developmental programming processes that have been reviewed by us (3, 4) and others (5–7) before substantially contribute to differences in morbidity and mortality between individuals. The normal development of the kidney can be disturbed by multiple environmental factors, including intrauterine deficiencies by disturbed placental function or maternal malnutrition, prematurity, intrauterine and postnatal stress, intrauterine and postnatal overnutrition, as well as dietary dysbalances of macro- and micronutrients. Since developmental steps take place during unique developmental periods, timing, and duration of an adverse environment specifically impact on developmental programming. Adverse kidney programming increases the incidence of severe renal and cardiovascular sequels later in life. This includes arterial hypertension and associated end organ damage, the aggravation of inflammatory glomerular disease and the occurrence of end-stage renal disease. Specific “re-programming” interventions may mitigate or even prevent programmed disease. Consequently, our mini-review will address the following topics:

(1) Which developmental stages are especially vulnerable?
(2) What are the long-term sequelae of adverse renal programming?
(3) Which environmental factors may have impact on kidney development and what are potential mechanisms of developmental kidney programming?
(4) What are potential therapeutic “re-programming” interventions?

## Critical Developmental Periods of Renal Programming in Humans

In humans the pronephros begins to form around day 22, urine production starts after 10 weeks (8), and maximum renal growth occurs between 26 and 34 weeks of gestation (9). Around week 36, nephrogenesis is completed and the number of nephrons is determined (8, 10). In preterm infants, adaptation to extra-uterine conditions impairs nephrogenesis, and the children end up with fewer nephrons and a higher percentage of morphologically abnormal glomeruli (5, 6, 11, 12). In small for gestational age (SGA) fetuses, intrauterine renal growth is reduced compared to appropriate for gestational age controls (9). In both term and preterm infants, glomerular and tubular functions undergo further maturational changes during the first months of life (8, 13). In these vulnerable periods, babies are often exposed to nephrotoxic medication, such as non-steroidal anti-inflammatory drugs (14), antibiotics, or diuretics, during neonatal intensive care unit treatment (15, 16).

## Long-Term Sequelae of Renal Programming in Humans

### Blood Pressure and Loss of Kidney Function

Hypertension is the most important risk factor for cardiovascular events and mortality worldwide (17). Elevated blood pressure contributes to progression of renal insufficiency (18) and is a strong independent risk factor for end-stage renal disease (19). *Vice versa*, decreased renal function is associated with increased blood pressure and cardiovascular morbidity (20). Early detection of blood pressure elevation plays a major role in the prevention of end organ damage (21). Many studies, including a systematic meta-analysis of studies tracking blood pressure during life course, demonstrated that childhood blood pressure predicts blood pressure (22–24) and vascular end organ damage in adulthood (25). Abnormal birth weight, either low or high, increases the risk for blood pressure elevation and loss of renal function in a U-shaped manner (26–31). In SGA individuals, some studies demonstrated elevated blood pressure in childhood (32) or adulthood (33, 34) especially when rapid postnatal catch-up growth and later adiposity were present (35). Further risk factors include high maternal BMI (36) or elevated protein/carbohydrate ratios in maternal diet during pregnancy (37, 38), rapid postnatal weight gain (39), or being born large for gestational age (36, 40, 41).

### Proteinuria and Loss of Kidney Function

Several risk factors during early life predispose toward proteinuria and related decline of renal function. Accordingly, the prevalence of microalbuminuria among adults whose mothers had been exposed to the Dutch Hunger Winter 1944/45 was elevated (42). Chinese women born in the famine years 1959–1961 had a higher risk to develop more severe stages of proteinuria in their forties (43). Low birthweight itself is associated with elevated risks for albuminuria (OR, 1.81; 95% CI, 1.19–2.77), end-stage renal disease (OR, 1.58; 95% CI, 1.33–1.88), or low estimated glomerular filtration rate (GFR) (29, 44, 45). A birthweight-dependent decline in GFR may already be seen in childhood (29, 46, 47).

### Glomerular Disease and Inflammation

Furthermore, a number of studies have evaluated the association between perinatal risk factors and later glomerular disease. Thus, SGA individuals have a higher risk to experience steroid resistance and a more severe course in nephrotic syndrome (48, 49). In IgA-nephropathy, they develop arterial hypertension and glomerulosclerosis more often (50).

## Mechanisms of Renal Programming

**Figure 1:**
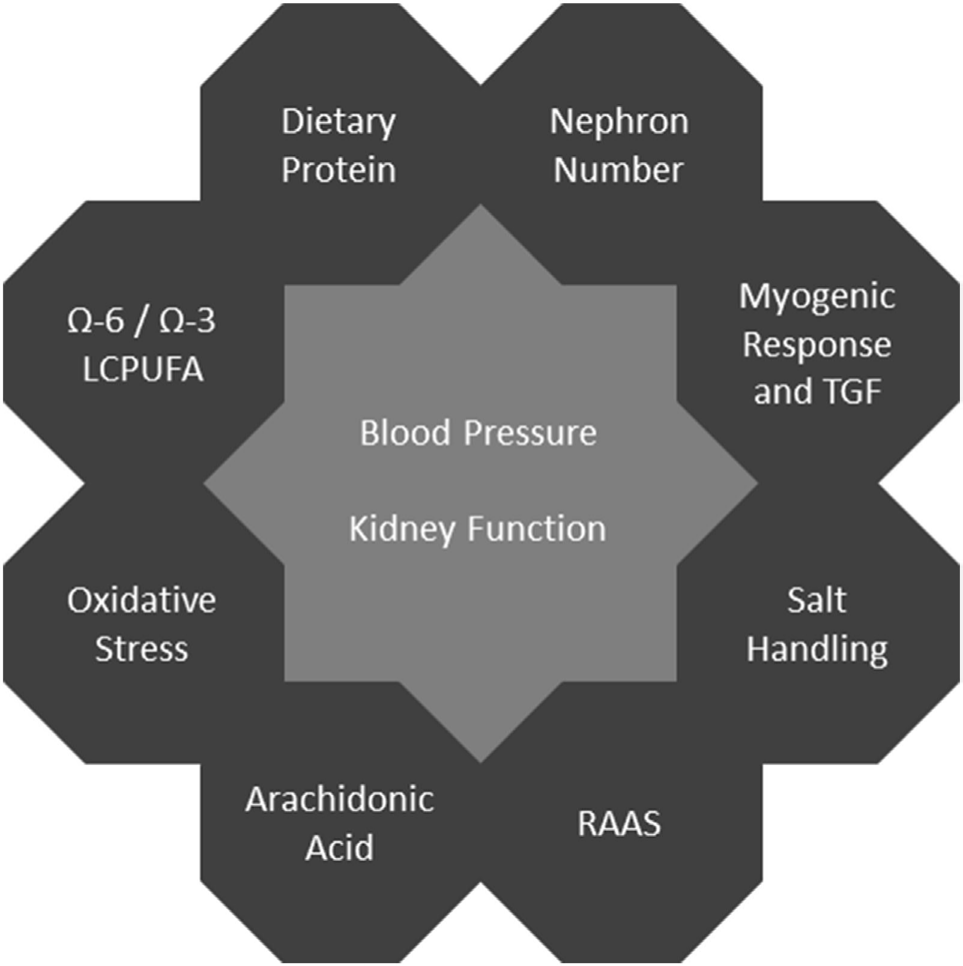
Programming mechanisms affecting blood pressure and kidney function.

In human studies, it is difficult to establish mechanistic links in the field of developmental programming since there usually is a large delay between an adverse event and the related clinical phenotype. This makes it very challenging to distinguish the underlying causes from multiple modifying factors. Thus, animal models providing the possibility of equalized postnatal conditions and specific interventions are especially valuable. In rodents, kidney development during the early postnatal period corresponds to the third trimester in humans (10). For an overview of mechanisms see Figure 1.

### Nephron Number

Nephron number in humans ranges from ~200,000 to >2.5 million nephrons per kidney (51). The well-known hypothesis of Brenner et al. linked decreased glomerular number with increased glomerular size, hyperfiltration, hypertension, and progressive glomerular injury (7, 52). Nephron number positively correlates with birth weight (53, 54) and is reduced after low-protein (LP) diet throughout pregnancy (55–59), utero-placental insufficiency (60–62), intrauterine glucocorticoid exposure (63), preterm birth (11, 64), and oxidative stress (65). In addition, a diet deficient in vitamin A (58, 66), zinc (67), or iron (68) is associated with low nephron count. Finally, nephrons get lost with age (69). Interestingly, low nephron number in young individuals is not necessarily associated with hypertension (70, 71). Thus, modulating factors such as early hyperalimentation and aging processes certainly have an impact on renal outcome in individuals with low nephron count (71, 72).

### Renin–Angiotensin–Aldosterone System (RAAS)

Dysregulation of all or single components of the RAAS system may severely impair renal development (73, 74). Both activating and deactivating effects on the RAAS can induce a vicious circle of persisting hormonal dysbalances which may finally contribute to the development of arterial hypertension and renal failure.

In the fetal and perinatal period, downregulation of the RAAS has been identified as a relevant mechanism. In neonatal rats after LP diet during gestation, renal renin and angiotensin II levels (75) as well as angiotensin II receptors type 1 (AT1R) and 2 (AT2R) protein expressions (76) were reduced. Similarly, renal AT2R gene and protein expressions were reduced in fetal rats after prenatal caffeine exposure (77). Fetal angiotensin II levels in plasma were decreased after maternal high-salt diet in sheep (78).

Later in life, most environmental influences during early childhood end up with a RAAS activation. Adult rat offspring from the LP diet model showed elevated blood pressure (59), increased AT1R expression (79, 80) and elevated plasma angiotensin-converting enzyme (ACE) activity going along with slightly elevated angiotensin II levels (81). When challenged with angiotensin II infusion, adult LP offspring reacted with a greater decline in GFR than controls (80). In another LP study, there were more angiotensin II-positive cells in the cortical tubulointerstitium of adult offspring (82). Offspring from diabetic mothers had marked upregulation of angiotensinogen (AGT) and AT1R gene expression as well as increased ACE:ACE2 mRNA ratio (83). Some environmental influences induce RAAS activation already in fetal life. Thus, ovine offspring exposed to high salt during gestation presented with increased gene expression of AGT, ACE, AT1R, and increased ACE:ACE2 and AT1R:AT2R mRNA ratio (78). In the human situation, plasma renin concentrations were elevated in umbilical veins of SGA infants, and birth weight was inversely associated with circulating aldosterone concentrations (84). Treatment of human proximal tubule epithelial cells with palmitic acid demonstrated susceptibility to nutritional factors, as it induced intracellular endoplasmic reticulum (ER) stress and increased angiotensin II concentrations in cell medium. Co-treatment with AT1R-blocker or renin-inhibitor prevented ER stress (85).

### Renal Sodium Transport

In rats, LP nutrition or dexamethasone treatment during gestation both resulted in an upregulation of the bumetanide-sensitive Na-K-2Cl cotransporter and of the thiazide-sensitive Na-Cl cotransporter in the offspring (86, 87). Adult offspring exposed to LP nutrition during gestation and lactation presented with a reduced diuretic response after a single dose of furosemide (88). After prenatal dexamethasone treatment, proximal tubule Na/H exchanger protein expression was increased, going along with an increase in proximal tubule sodium and volume reabsorption (86, 89). Sodium uptake in renal proximal tubule cells from adult male sheep was enhanced after prenatal betamethasone exposure (90). In rat offspring exposed to experimental utero-placental insufficiency (91) or maternal diabetes (92), sodium-dependent hypertension was observed.

### Vasomotor and Endothelial Function

Another interesting aspect is the maturation of vascular smooth muscle function and small artery resistance regulation. Sympathectomy suppresses maturation of the gene program involved in small artery resistance regulation (93). Intrauterine and perinatal stress could, therefore, have a major impact on vascular tone regulation. In addition, vasomotor function can be impaired by perinatal hyperoxia (65) and LP diet (94). Endothelial dysfunction has also been described after intrauterine deficiency and may add to hypertension and glomerular damage (95).

### Myogenic Response and Tubuloglomerular Feedback (TGF)

An impaired myogenic response as well as a disturbed TGF are important contributors to glomerular damage in diabetic and hypertensive nephropathy (96). An altered myogenic response has been described in intrauterine growth-restricted (IUGR) neonates, which may be beneficial postnatally, but harmful in the long run (97). The TGF mechanism matures during fetal life and could, therefore, be susceptible to programming *in utero* (98). However, no study has examined the specific consequences of disturbed intrauterine environment for TGF function.

### Epigenetic Mechanisms

The molecular details of kidney development have been extensively studied (99, 100). Altered DNA methylation, histone modification, and other mechanisms modifying the renal transcriptome may significantly impair renal organogenesis and predispose toward renal disease which has lately been reviewed in detail (101). In this context, it is important to separate epigenetic changes during kidney disease (102) from epigenetic changes during early life leading to “programmed” disease. So far, there is little evidence that single, kidney-specific epigenetic alterations during early life might actually cause renal disease later on. Candidate genes would be all genes which are activated during specific developmental windows. Pax-2, for example, is essential for kidney development, ontogenetically regulated and can be reactivated in repair processes after acute kidney injury (103, 104). Global alterations of methylation associated with hypertension were observed after significant periconceptional deficiency of B vitamins and methionine (105). Thus, nutritional modifications may induce temporary or permanent epigenetic alterations that certainly have the potential to modulate kidney disease.

### Oxidative Stress

Oxidative stress and inflammation are major contributors to vascular remodeling and hypertension (106). In LP (94, 107) and maternal smoking models (108), it was shown that oxidative stress during critical developmental steps may significantly contribute to renal susceptibility toward disease. In addition, both IUGR offspring after global undernutrition of the dam (109) and after high-fat died during gestation and lactation (110) showed increased oxidative stress and elevated blood pressure later in life. Reduction of oxidative stress during early life can prevent programmed hypertension and renal damage (94, 107, 108).

### Metabolism

Rapid postnatal weight gain and early life obesity have been associated with adverse renal outcome (111, 112). Interplay between adiposity, leptin, and insulin resistance with RAAS regulation and sympathetic activity has been described (113, 114). Early postnatal overfeeding in rats by litter size reduction induced increased early postnatal weight gain and was associated with increased blood pressure, glomerulosclerosis, and proteinuria in adulthood (71). In a similar study, postnatal overfeeding resulted in decreased GFR, increased proteinuria and increased deposition of collagens. On the molecular level, intrinsic renal leptin resistance could be demonstrated (115). Dysregulation of renal leptin and Akt/AMPKα signaling associated with increased renal matrix deposition could also be shown in overweight offspring from mothers fed a high-fat diet during gestation and lactation (116). Maternal LP nutrition during rat gestation persistently decreased the expression of renal 11β-hydroxysteroid dehydrogenase type 2 (11β-HSD2) (117, 118) and increased the expression of the renal glucocorticoid receptor in the offspring (118). The same was shown for sheep offspring exposed to temporary maternal calorie restriction (119).

### Arachidonic Acid Metabolism Pathway

Finally, there is evidence that the arachidonic acid metabolism pathway could be involved in the development of programmed hypertension (120, 121). 20-hydroxyeicosatetraenoic acid (20-HETE), a metabolite of arachidonic acid, contributes to the normal myogenic pressure response. Physiologically, arachidonic acid is released from cell membranes by phospholipase A2, converted to 20-HETE, which then adds to vasoconstriction of the afferent arteriole (96, 122). However, 20-HETE has also been linked to systemic hypertension and endothelial dysfunction in rats (123). Further arachidonic acid metabolites like Cox-2 derived prostaglandins contribute to counter regulatory vasodilation of the afferent arteriole after TGF-mediated vasoconstriction (124) and oxidative stress in the kidney (125), and therefore modulate intraglomerular pressure and GFR as well as renal inflammation (82). Thus, nutritional intake of arachidonic acid may significantly affect blood pressure, kidney function, and kidney survival.

## Potential Therapeutic “Re-Programming” Interventions

The ultimate goal of all research on programmed disease is to develop preventive strategies. So far, the number of studies on re-programming interventions is still limited and mainly restricted to dietary or anti-oxidative approaches.

### Early Dietary Interventions

Data on nutritional interventions are available from both animal and human studies. A meta-analysis showed slightly, but significantly lower blood pressure in infants, children, and adolescents who were breast fed during infancy compared to those being formula fed (126). Micronutrient (127), calcium (128), vitamin A (129, 130), and iron (131) supplementation during pregnancy as well as long-chain polyunsaturated fatty acid (LCPUFA) supplementation in infant formula (132) may be beneficial to renal outcome.

In detail, children of women receiving a multiple micronutrient supplementation during the second and third trimesters of pregnancy were heavier and had lower systolic blood pressure during infancy (127). Calcium supplementation from the 20th gestational week until delivery lowered systolic blood pressure in children aged 7 years, with a stronger effect when children were overweight (128). Supplementation of iron and folate until the end of pregnancy in rural Bangladesh caused a slightly decreased diastolic blood pressure and a slightly increased GFR in infants at the age of 4.5 years when started at the ninth, but not when started at the 20th gestational week (131). Another dietary intervention with micronutrient supplementation in malnourished pregnant Nepalese women until 3 months postpartum showed that folic acid or the combination of folic acid, iron, and zinc reduced the risk of microalbuminuria, but not blood pressure in the children aged 6–8 years (133). The effects of retinoic acid have mainly been studied in animals. Decreased availability of retinoic acid induced by down-regulated vitamin A metabolism after previous overexposure to vitamin A strongly impairs metanephric kidney development, which can be restored by adequate retinoic acid supplementation (129). In rat offspring exposed to LP diet of the dam during pregnancy, a single injection of retinoic acid to the dam at midgestation increased postnatal nephron number at 4 weeks of age (130). Postnatal administration of retinoic acid in preterm baboons, however, did not alter kidney growth or nephron number, presumably because the timing of the intervention was chosen too late (134).

Long-chain polyunsaturated fatty acid supplementation with arachidonic acid and docosahexaenoic acid (ratio 2:1) in infant milk formula (IF) during the first 4 months of life lowered blood pressure at 6 years of age compared to IF without LCPUFAs. Blood pressure of children fed LCPUFA-IF was similar compared to breast fed children (132). A diet sufficient in ω-3 PUFAs reduced blood pressure levels compared to a diet almost free of ω-3 PUFAs in TGR(mRen-2)27 rats which have high angiotensin II levels (135). Finally, the importance of the amino acid composition was demonstrated. Addition of glycine to maternal LP diet throughout gestation normalized body weight and blood pressure at 4 weeks of age in rat offspring, whereas alanine or urea had no effect (136).

### Anti-Oxidative Substances

Re-programming interventions with anti-oxidative substances have only been performed in animals. Supplementation of maternal LP diet with anti-oxidative (ACH09)-derived polyphenols extracted from grape skins reduced signs of renal oxidative stress in the offspring on the first postnatal day and attenuated the adverse effects of maternal LP diet on glomerular number and maturity (107). Administration of a lipid peroxidation inhibitor along with LP diet in gestation reduced prenatal oxidative stress and prevented programming of elevated blood pressure, enhanced vasoconstriction after angiotensin II administration and reduced vasodilation after sodium nitroprusside administration in adult animals (94). Similarly, treatment of previously malnourished dams with α-tocopherol during lactation prevented the development of hypertension in the offspring. In addition, upregulated angiotensin II levels and down-regulated Cox-2 expression in the tubulointersititum were brought back to control levels and oxidative stress as well as macrophage infiltration was prevented. However, treatment of control dams with α-tocopherol resulted in arterial hypertension of the offspring (82).

## Conclusion and Future Directions

The concept of “developmental origins of health and disease” highlights the interrelationship between environmental factors throughout life and differences in morbidity and mortality between individuals. Chronic kidney disease affects more than 10% of the population (1). High blood pressure, childhood underweight, and suboptimal breastfeeding are among the top risk factors contributing to global burden of disease (137). Prematurity, IUGR, overweight in early life, and other conditions have been associated with the development of arterial hypertension, proteinuria, and decline of renal function. Around 11% of all live-born infants worldwide are born preterm (138). IUGR is seen in 3–7% of all pregnancies (139). During childhood, 5–6% of girls and 7–8% of boys become overweight (140). Thus, renal programming is not a rare phenomenon but affects large parts of the population. Further studies that broaden our understanding of renal programming mechanisms are needed to ultimately develop preventive strategies. Targeted re-programming interventions in animal models focusing on known mechanisms will contribute to new concepts which finally will have to be translated to human application. Early nutritional concepts with specific modifications in macro- or micronutrients are among the most promising approaches to improve future renal health.

## Author Contributions

EN performed the majority of literature research, and designed and wrote the review. K-DN contributed to literature research, and designed and wrote the review. JD and LW contributed to literature research and writing. All authors revised and approved the review.

## Conflict of Interest Statement

The authors declare that the article was written in absence of any commercial or financial relationships that could be a potential conflict of interest to the topic.
